# Tip-induced nanoreactor for silicate

**DOI:** 10.1038/srep14039

**Published:** 2015-09-14

**Authors:** Ming Gao, Liran Ma, Yong Liang, Yuan Gao, Jianbin Luo

**Affiliations:** 1 State Key Laboratory of Tribology, Tsinghua University, Beijing, 100084, China

## Abstract

Nanoscale scientific issues have attracted an increasing amount of research interest due to their specific size-effect and novel structure-property. From macro to nano, materials present some unique chemical reactivity that bulk materials do not own. Here we introduce a facile method to generate silicate with nanoscale control based on the establishment of a confined space between a meso/nanoscale tungsten tip and a smooth silica/silicon substrate. During the process, local water-like droplets deposition can be obviously observed in the confinement between the Si/SiO2 surfaces and the KOH-modified tungsten tip. By the combination of in-situ optical microscopy and Raman spectroscopy, we were able to take a deep insight of both the product composition and the underlying mechanism of such phenomena. It was indicated that such nanoreactor for silicate could be quite efficient as a result of the local capillarity and electric field effect, with implications at both nano and meso scales.

Recently, with the rapid development of high-quality small-scaled techniques and the unlimited possibilities of fabricating micro structures, people are moving the spotlights on researches from macro scale to meso/nano-scale. As a science with objects of smallest dimension, a large amount of unique properties that bulk scale do not possess have been observed in the research of nanotechnology. These not only depend on the size, shape, molecular structure, but also the pressure and temperature of the environment. A considerable amount of materials have already been demonstrated that when they decrease to a critical size, their electrical conductivity^1^, melting point^2^, chemical reactivity^3^ and magnetic permeability^4^ will be completely different from that at macro scale. Meanwhile, in the nano-sized regime, significant effect would be initiated due to closely confined space, such as the quantum-mechanical confinement effect in nanoelectronics, in which the energy of delocalized electrons increases with decreasing size^5^, and the titanate nanotubes (TNTs)^6^, which can offer a special nano-confinement location. Other scientific fields, like biochemistry^7^ and tribology^8^,^9^, also present a great interest and bright foreground in nano-sized issues.

In the field of chemistry, a lot of close attention has been paid to nanoscience, particularly the newborn nanometer sized reactor. Over the past years, the so-called nanoreactors^10^, performing a coupling of reactions in time by confining reagents and catalysts within a nanospace, have presented great potential in improving chemical transformation. So far, several advantages have emerged using theses nanoconfined cell, including reducing reaction time, minimizing amounts of reagents and obtaining fresh productions. For catalytic reaction systems, functional nanoreactors are widely used. They are mostly realized basing on hollow spheres, especially mesoporous silica hollow spheres. Song *et al.* fabricated a composite nanoreactor with mesoporous silica hollow spheres and Pd nanoparticles inside, which shows remarkable activity for Suzuki cross-coupling reactions^11^. Lee *et al.* introduced an ideal nanoreactor system comprising gold cores and silica hollow shells with empty inner space for the reduction of p-nitrophenol^12^. Nanophase-separated amphiphilic networks also have been demonstrated to possess the ability to stabilize and enhance the catalytic activity of enzymes in organic solvents^13^.

However, a bridge between nanoreactors and inorganic chemistry has not been built up because of lacking practical application. Furthermore, these nanoreactor require complex fabrication process and extremely sophisticated manipulation, which limits its development in industry. In fact, with a view to creating and exploring new chemical productions and novel physical phenomena, nano scientific effort should be carried out to seek different disciplines. To achieve this goal, a facile method has been developed to create a nanometer-size cell with an electrochemical etching tungsten tip ranging from tens nanometer to hundreds nanometer. The technique first applied nanoreactor to organic materials, inducing a specific chemical reaction without catalyst at room temperature, which generally occurred demanding high thermal energy. Meanwhile, instantaneous springing out of water droplets was observed along with the reaction process.

Herein, a micro-manipulator was used to control a nanoscale tungsten tip while reciprocating sliding occurring between the tip and substrate. During the experimental procedure, droplets of “water-like” precipitation were observed in the confined space. The compositions of the products and the underlying mechanism were well investigated with various characterization methods. It has been speculated that certain chemical reaction between KOH residual on the tip and Si/SiO_2_ surface has been induced and facilitated by the nanoscale reactor between tip and substrates. The major breakout in our work is to produce silicate with nanoscale control even at room temperature. The study made further effort to simplify the concept and fabrication of nanoreactor, introducing it into organic materials. What’s more, it also provided a unique sight for silicate products industry. In our future research, large amount of work will be tended to expand the area of nanoreactor application, trying to discovering more similar reactions and phenomena.

## Methods

The tungsten tips, terminated with a spherical cap of different radius, were prepared by etching electrochemically in 2 M potassium hydrate (KOH) under 15-V alternating voltage for 90 s in the first stage then sharpened under 2-V alternating voltage for various time.

The substrate samples used in the experiment were N-type (38–50 Ωcm) Si (111) wafers and Si (100) wafers covered by 300 nm SiO_2_ after thermal oxidization. Prior to the operation, the substrate samples were immersed into the acetone solution and alcohol solution for 30–60 s separately in order to eliminate organic contaminants, followed by an extensive rinse with ultrapure water. After the cleaning procedure, the samples were completely dried by N_2_ gas flow.

**Figure 1:**
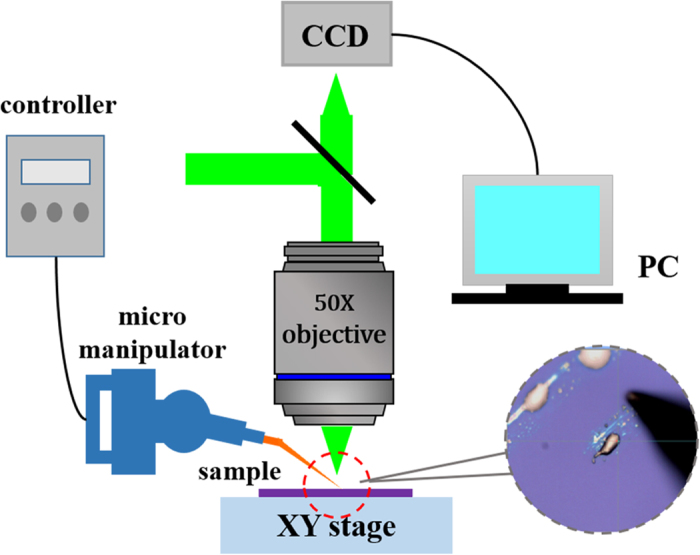
Schematic of experimental setup. This Figure is drawn by Ming Gao and Yong Liang.

In a typical experiment, the temperature and relative humidity in the environment is two of the key factors. We conducted it in a near-constant temperature and humidity room. The temperature was kept at 20 ± 2 °C and the relative humidity was maintained around 40 ± 5%. The whole manipulation was performed under an optical microscope with a 50× long working distance microscopic objective. The scattered Raman signal was collected in a backscattering configuration through the objective, filtered, and then dispersed onto a liquid-nitrogen-cooled CCD camera through a single grating spectrometer. As shown in Fig. 1, the motion of the tungsten tips was well controlled by a micromanipulator (Kleindiek, MM3A), then it touched slightly on the substrate surface, with a slow sliding rate of 5 μm/s. The products were observed with scanning electron microscopy (SEM), related characterization was carried out on energy dispersive X-ray (EDX) and Auger Electron Spectroscopy (AES), and the molecular structure was analyzed using Raman Spectroscopy.

## Results

### The electrochemical etching of tungsten tips

**Figure 2:**
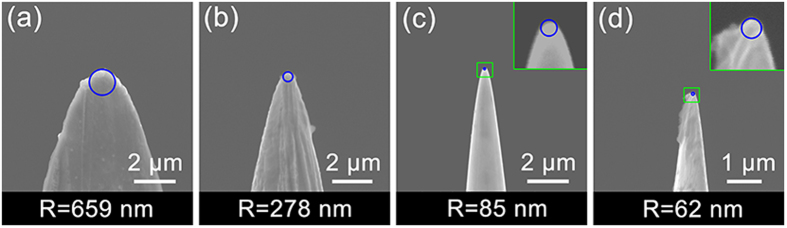
SEM images of the tungsten tips sharped via electrochemical etching. The tungsten wires were etched in 2 M potassium hydrate (KOH) solution under 15-V alternating voltage for 90 s in the first stage then were applied under 2-V alternating voltage for various time, for 10 s, 20 s, 40 s, and 60 s from panel (**a**) to panel (**d**). The radius of curvature of each tip is marked in the graph.

It has been confirmed that the formation of meso/nano-sized tungsten tips mainly depends on the voltage and etching time during the electrochemical corrosion. To obtain different radius of curvature, etching time under 2-V alternating voltage has been controlled. As shown in Fig. 2, from panel (a) to panel (d), with the etching time increasing gradually, the radius of tip decreased (from hundreds of nanometer to tens of nanometer). In addition, from the SEM images it can be inferred that some residual KOH from the etching solution has adhered on the head of tips after electrolytic corrosion.

### Silicate produced in the nanoreactor

**Figure 3:**
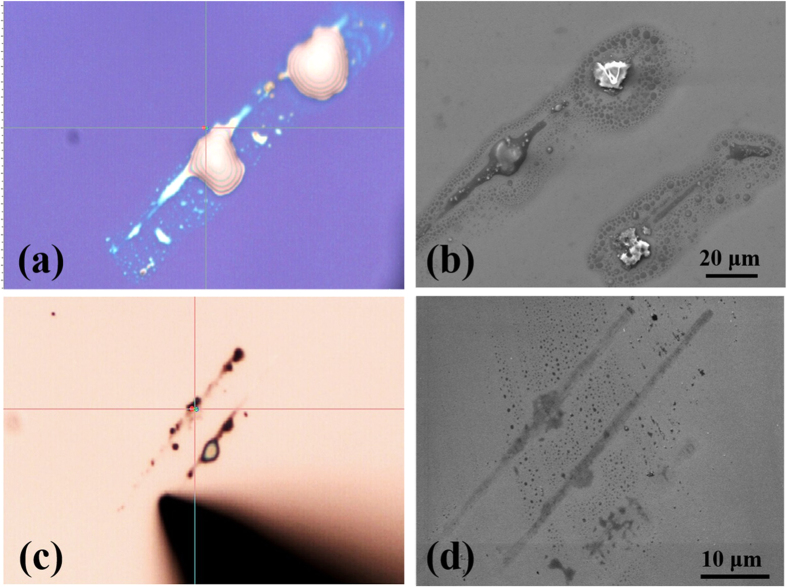
The optical microscopic images and SEM graphs of the precipitation on substrate. The experiments were operated under room temperature, and the humidity was around 45%. The radius of the tungsten tip used here was 278 nm for SiO_2_ substrate and 85 nm for Si substrate. Panel (**a**–**b**) were the images of the SiO_2_ substrate and part (**c**–**d**) were the images of the Si substrate.

The optical microscopic images (panel a and c) and SEM images (panel b and d) of products were directly shown in Fig. 3. For part 3a and 3b, the substrate was the Si wafer covered with 300 nm SiO_2_, while for part 3c and 3d, the substrate was the N-type Si wafer. The initial status of precipitations were water-like droplets on both substrates, after deposited in air at room temperature for one day, the products dried out. Here, the radius of the tungsten tip was 278 nm for SiO_2_ substrate and 85 nm for Si substrate respectively.

Here the tip size is one of the key factors for this induced chemical reaction. Tungsten tips with different radius were obtained through electrochemical corrosion in KOH solution, as displayed in Fig. 3. A series of experiments had been carried out with tips of different size on each surface. The droplets deposition can be only observed when the radius of curvature of tips was less than ~500 nm. On the other hand, we also found that when the size of the tips ranged from tens to hundreds nanometers, bigger tips can produce more product.

## Discussion

**Figure 4:**
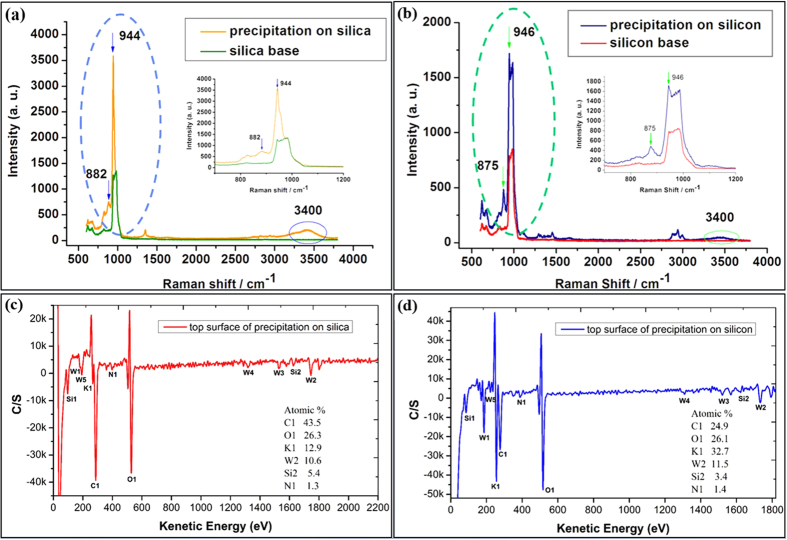
The collected Raman signal and AES of products. The Raman experiments were carried out under room temperature and the humidity was around 45%. Panel a is Raman spectra of silica base and its precipitation shown in Fig. 3b, while panel (**b**) is Raman spectra of silicon base and its precipitation shown in Fig. 3d. Several significant peaks were marked in the graph. The AES spectrum was collected from the top surface of products. Part a presented the signal collected from the precipitation on silica surface displayed in Fig. 3b and part (**b**) presented the signal collected from the precipitation on silicon surface displayed in Fig. 3d.

In order to further investigate the underlying mechanism of this phenomenon, we first characterize the water-like products with Raman spectra and AES. Panel a and b in Fig. 4 shows the results of Raman spectra collected right after the precipitation sprung out. To make a deep and comparative analysis, the Raman spectra of silica and silicon substrate has also been introduced. The inset part in each panel is the local Raman spectra ranging from 700 to 1200 cm^−1^. Comparing the spectra of base and products, we found that the peaks near 880 cm^−1^, 950 cm^−1^, 3400 cm^−1^ came out for both substrates. As reported in the previous researches, the band near 880 cm^−1^ is assigned to the Si-O stretching^14^ and the band near 950 cm^−1^ is assigned to the Si-OH stretching^15^, while 3400 cm^−1^ presented the O-H stretching in liquid water^16^. It was suggested that some kind of silicate had been produced during the chemical reaction between the substrate surface and the KOH left on the tip, which is quite different from macroscopic issues. In general, KOH solution can hardly react with Si or SiO_2_ at room temperature. As reported in the past documents, the etching rates between KOH and Si were measured at 3000 ~ 4000 Å/min at 62 °C^17^, while at room temperature these rates become unnoticeable. For the chemical reaction between KOH and SiO_2_, the temperature usually was set to 1300 °C. It can be inferred that these chemical reactions have been effectively enhanced by the tip-induced nanoscale space.

The result of AES spectrum helps us to further confirm the above hypothesis by providing the elements composition of products. To exclude the influence of substrate, we only collect the AES signal from the top surface of each precipitation, which was about 10 nm deep. As shown in Fig. 4c,d, from the AES spectrum, we can understand clearly that elements like K, O, C, W, Si, and N were concluded in the products, indicating that potassium silicate had been generated. The resource of W element was the potassium tungstate remaining on the tip, which has been produced during the electrochemical etching process.

**Figure 5:**
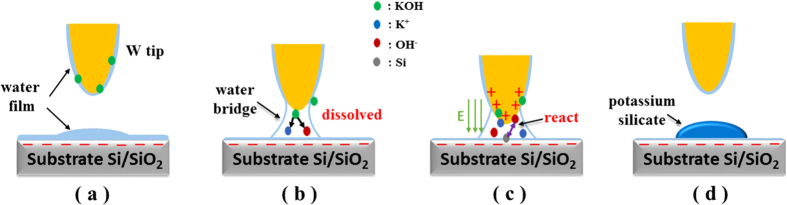
Schematic sketch of the “water-like” droplets precipitation process.

To illustrate the precipitation procedure in the nanoreactor, we develop a simple analytical model to describe the presence of the water-bridge that forms between the tip and substrate and to analyze the molecular mechanisms of such nanoscale transporter, as displayed in the Fig. 5. In part a, both tip and substrate were assumed to be hydrophilic, they were wetted by a thin water layer in equilibrium with the ambient humidity. Here the relative humidity was set up to be 40 ± 5%. In addition, SiO_2_ surface immersed in water is known to own a negative surface charge destiny, primarily through the dissociation of terminal silanol groups^18^. As the tip approaching, a water bridge between the substrate surface and tip was formed. This water meniscus played an important role here as a nanometer-sized cell for chemical reaction. At first stage. it served as a tiny capillary in which KOH can be dissolved into K^+^ and OH^−^, then these metal ions can be transported from tip to substrate surface, as shown in part b. Once the tip moved into the range of electric double layer of SiO_2_ surface, positive charge would be stimulated at the tip top. In this case, an extremely high electric field has been induced. By Gaussian theorem, the electric field intensity is inversely proportional to radius squared. Thus as the decreasing of the tip dimension, higher electric filed energy was assembled in the nanoconfine area. That is the reason why smaller tips here can promote the generation of silicate.

Once the intensity of the electric field reached to a specific point, it can provide enough power to increase the Gibbs energy, which can help KOH and Si/SiO_2_ obtain activation energy required, leading to the reaction in the nanoscale space, even at room termperatue^19^. So the second step was, as displayed in part c, under the effect of electric field, OH^−^ was absorbed onto the surface of substrate, along with the occurrence of chemical reaction and the formation of silicate. For Si substrate, the KOH etching generally involves at least three reactants: OH^−^, H_2_O and silicon^20^. The reaction may be described by equation (1)^21^:


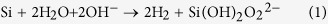


Similarly, SiO_2_ substrate also occurred a chemical etching, and the reaction can be simplified to equation (2)^22^:


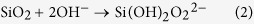


Here, for both substrates, a nanoreactor was created, in which above reactions have been induced, with Si(OH)_2_O_2_^2−^ as the final products, confirmed by the Raman spectra and EDX, AES results. As a strong water-absorbing silicate, Si(OH)_2_O_2_^2−^ can strongly grasp the water from air. Furthermore, potassium tungstate generated in the etching experiment has been induced via the water bridge. Due to the absorbency property owning by produced potassium silicate and potassium tungstate, ambient water vapor was quickly absorbed, leading to the observed deposition of “water” droplets under optical microscopy.

### Outlook

In conclusion, a simple system aimed at creating confined space induced by a nanoscale tungsten tip has been designed. We are surprised to observe a phenomenon during the experiments — springing out of big “water-like” droplets. It has been speculated that the H_2_O-bridge between the tip and substrate provides a passage for the hydration of K^+^ ions and OH^−^ ions. In the nanoreactor induced by the tip, an extremely high concentration area filled with electric field and OH^−^ ions has been created, with a local high-pressure and high-temperature area. These conditions would facilitate the reaction between OH^−^ and Si/SiO_2_, leading to generation of silicate, which serves as the source of droplets. Our new findings in this work open up fascinating views to the researches on the nanoscience.

## Additional Information

**How to cite this article**: Gao, M. *et al.* Tip-induced nanoreactor for silicate. *Sci. Rep.* **5**, 14039; doi: 10.1038/srep14039 (2015).
